# Determinants of Family Empowerment and Complementary Feeding Quality: Evidence from a Transcultural Care Framework

**DOI:** 10.3390/healthcare13172237

**Published:** 2025-09-08

**Authors:** Bayu Prabowo, Ratna Wardani, Agusta Dian, Suwarto Suwarto

**Affiliations:** 1 Graduate Programme, Universitas Strada Indonesia, Kediri 64123, Indonesia; bay_fj40@yahoo.com (B.P.); ratnawardani61278@gmail.com (R.W.); 2 Nursing Management, Universitas Strada Indonesia, Kediri 64123, Indonesia; agustadian85@gmail.com; 3 Faculty of Agriculture, Universitas Sebelas Maret, Surakarta 57126, Indonesia

**Keywords:** stunting prevention, complementary feeding, family empowerment, transcultural nursing, structural equation modeling, maternal education, cultural factors

## Abstract

**Background:** Stunting remains a major public health issue globally and in Indonesia, often linked to inadequate complementary feeding, cultural practices, and limited family empowerment. Objective: This study aimed to develop and evaluate a family empowerment model based on transcultural care theory to improve quality and prevent stunting among children aged 6–24 months. **Methods:** A cross-sectional explanatory survey was conducted among 324 mother–child pairs from 11 primary healthcare centers in Kediri, East Java. Data were collected using a validated questionnaire covering demographic, educational, technological, economic, and cultural factors, as well as family empowerment and quality. Structural Equation Modeling with Partial Least Squares (SEM-PLS) was applied for hypothesis testing and model development. **Results:** The model showed moderate explanatory power (R^2^ = 0.223 for family empowerment; R^2^ = 0.115 for complementary feeding quality). Demographic, educational, technological, economic, and cultural factors significantly influenced family empowerment (*p* < 0.05), which in turn had a strong positive effect on quality (β = 0.340, *p* < 0.001). Family empowerment mediated the relationship between these factors and quality. Key contributors included knowledge, technology access, income level, and cultural practices. **Conclusions:** The proposed transcultural care-based family empowerment model effectively improves quality. Strengthening health education, supporting community health volunteers, and integrating culturally sensitive practices, such as encouraging paternal involvement and shared meals, should be prioritized in stunting prevention programs. The model may be adapted for use in similar community settings to enhance program effectiveness.

## 1. Introduction

Stunting is a growth and developmental disorder experienced by children due to poor nutrition, recurrent infections, and inadequate psychosocial stimulation [1]. It is a syndrome of linear growth failure that serves as an indicator of various pathological conditions associated with increased morbidity and mortality, loss of physical growth potential, and impaired neural development and cognitive function, as well as an elevated risk of chronic diseases in adulthood [2]. This condition results from chronic nutritional deficiencies and recurrent infections during the first 1000 days of a child’s life, a critical developmental window in which families play a central role [3]. Ensuring adequate nutrition, preventing infections, and maintaining access to healthcare during this period requires strong family empowerment. Positioning families as active agents in this window of vulnerability underscores the rationale for a family empowerment model, which aims to strengthen parental capacity and engagement in stunting prevention.

Stunting is a nutritional problem that requires urgent attention. In 2020, approximately 149.2 million children worldwide, under the age of five, suffered from stunting. It is the most common nutritional disorder among children in Southeast Asia, affecting about 25% of children under five years old [4]. Indonesia ranks as the country with the third-highest prevalence of stunting in Southeast Asia, with a prevalence of 36.4% between 2005 and 2017 [1,5]. According to the Indonesian Nutritional Status Survey (SSGI), the prevalence of stunting in Indonesia in 2022 was 21.6% [6]. The highest incidence of stunting occurs in children under five years of age, with 18% categorized as severe stunting [7]. Stunting has long-term impacts on both individuals and society, including impaired cognitive and physical development, reduced productive capacity and health status, as well as an increased risk of degenerative diseases such as hypertension and diabetes [8]. If the current trend continues, it is projected that 127 million children under the age of five will experience stunting by 2025. Therefore, stunting prevention is essential to mitigate both short- and long-term adverse effects and to achieve the WHO target of reducing the number of stunted children by 2025 [3].

Prevention strategies require comprehensive interventions targeting all risk factors for stunting, including nutrition education for key stakeholders (community health volunteers, mothers of toddlers, pregnant women, and prospective mothers), iron supplementation for pregnant women, provision of supplementary foods for underweight children, complementary feeding programs, basic immunization, vitamin A supplementation, and the establishment of learning groups facilitated by healthcare workers, as well as ensuring there are proper facilities, access to clean drinking water, and adequate sanitation [9,10].

The Indonesian government has implemented various policies to address stunting; however, several challenges persist during implementation. Research by Herawati & Sunjaya (2022) [11] reported significant barriers at the village, sub-district, and district levels across Indonesia in executing government policies to reduce stunting. Common obstacles include lack of commitment, insufficient staff capacity, and weak coordination, resulting in suboptimal program implementation and an ongoing burden on the frontline healthcare sector.

One preventive intervention that can be applied in Indonesia is family empowerment. A study involving 150 mothers in Surabaya demonstrated that family empowerment through early stunting detection training significantly influences stunting prevention in children [12]. Family empowerment refers to efforts aimed at fostering knowledge, skills, awareness, and resource capabilities to maintain and improve health status, achieve positive life control, and enhance quality of life [13].

A theoretical approach that incorporates cultural factors in the child nutrition process to prevent stunting is transcultural care. Transcultural care is a grand theory that integrates sociocultural factors into health-related decision-making [14,15]. The strength of this approach lies in its ability to provide culturally specific and universal practices for promoting health and well-being, or for assisting individuals in coping with unfavorable conditions, illness, or death in a culturally meaningful way [16]. Delivering culturally sensitive or transcultural care improves patients’ overall well-being, fosters a sense of respect and dignity within healthcare services, enhances patient satisfaction with healthcare providers’ behaviors, and ultimately contributes to better health outcomes [17].

Transcultural care, developed by Madeleine M. Leininger, addresses eight dimensions: demographic, educational, technological, social, political and legal, economic, religious, and cultural factors [14,16]. This model has been widely applied in Indonesia to improve child nutrition. For instance, Hidayat & Uliyah (2019) [18] reported that culturally based nursing care models could be implemented for families with malnourished toddlers from the Madurese ethnic group, who traditionally lack adequate childcare practices. Similarly, research by Tat et al. (2020) [19] found a significant association between health technology utilization, family philosophy, family cohesion, cultural and lifestyle factors, family economy, and parental education with the nutritional status of toddlers in Kupang.

Stunting is caused by the interaction of multiple complex factors operating not only at the individual level but also at household and community levels. Therefore, interventions must adopt a multi-level approach to address determinants across these levels [7]. Sociocultural factors are among the key risk factors for stunting in Indonesia; thus, there is an urgent need to develop community-based models that enhance knowledge and foster behavioral and cultural changes conducive to better nutritional practices.

In Indonesia, family cultural values strongly influence child nutrition, where parenting styles and the family nutrition climate can predict feeding practices and contribute to children’s nutritional outcomes [20]. However, existing studies on transcultural nursing in child nutrition mostly examine isolated factors and remain descriptive, without integrating them into a comprehensive family empowerment framework. This study addresses that gap by developing and testing a family empowerment model based on transcultural care theory to improve complementary feeding quality and support stunting prevention at the community level in Indonesia.

Therefore, the objective of this study is to evaluate the determinants of family empowerment and their effect on the quality of complementary feeding within a transcultural care framework. We hypothesize that demographic, educational, technological, economic, and cultural factors significantly influence family empowerment, and that family empowerment, in turn, has a positive impact on the quality of complementary feeding among children aged 6–24 months.

## 2. Materials and Methods

### 2.1. Study Design

This study employed a cross-sectional design using an explanatory survey approach.

### 2.2. Study Subjects

The sample of this study consisted of families with children aged 6–24 months in Kediri Regency, East Java, Indonesia. Data collection was carried out in the following 11 community health centers (Puskesmas) with the highest prevalence of stunting: Papar, Puh Jarak, Puncu, Kepung, Mojo, Semen, Tiron, Sambi, Tarokan, Badas, and Bendo. The study was conducted from 1 January 2025 to 1 May 2025.

The inclusion criteria for this study were as follows: (1) families with children aged 6–24 months who were identified as stunted; (2) residing in the Kediri Regency area; (3) nuclear families; and (4) parents who were literate, as determined by self-report of being able to read and understand the questionnaire. Families were excluded if they declined to participate or were not available during the data collection period. The focus on nuclear families was chosen to ensure homogeneity in caregiving practices and decision-making processes regarding child nutrition, which may vary considerably in extended family structures. While this increases internal consistency, we acknowledge that it may limit the generalizability of findings to households with extended family arrangements.

The sampling technique used was non-probability sampling with purposive sampling, in which participants were deliberately selected based on the above inclusion criteria to ensure the appropriateness of the sample.

The required sample size was determined using the following formula:

$$\;\;\frac{N}{1\;+\;N\cdot e^{2}}\;$$

where n = sample size, N = population size, and e = margin of error. With a known population of 1328 families, a 5% margin of error, and an alpha level of 0.05, the minimum required sample size was calculated to be 308 respondents. To strengthen the robustness of the study and account for possible non-response, the target sample size was increased, and data were ultimately collected from 324 families. Full details of the calculation are provided in Supplementary Materials S1.

### 2.3. Study Procedure

The researchers coordinated with stunting program officers at each community health center to validate the stunting patient data. After obtaining data on families with stunted children, respondents who met the inclusion criteria were selected to ensure an appropriate sample using purposive sampling.

The instrument used in this study was a structured questionnaire designed to assess the quality of complementary feeding (CF) provided to children aged 6–24 months. It was self-developed and modified by the researchers based on relevant variables and consisted of structured questions and statements, allowing respondents to mark appropriate responses (√). The questionnaire, distributed via Google Forms and WhatsApp, covered demographic, educational, technological, economic, cultural, and family empowerment factors, as well as complementary feeding quality (full version in Supplementary Materials S2). The complementary feeding index was adapted from Garg & Chadha (2009) [21] and modified to fit the dietary and geographical context of Indonesian communities, while also incorporating WHO-recommended indicators: timeliness of introduction, minimum meal frequency, minimum dietary diversity, and minimum acceptable diet. Reliability testing produced acceptable Cronbach’s alpha values for knowledge (0.804), technology utilization (0.709), collaboration (0.904), and complementary feeding (0.665). A validity and reliability test of the questionnaire was first conducted on 30 families with stunted children in September 2024, confirming the instrument’s validity and reliability. Following this, data collection was carried out with the assistance of 11 trained healthcare workers across 11 community health centers, under the coordination of the lead researcher to ensure efficiency, accuracy, and standardized procedures.

Data collection was initially planned through Google Forms distributed via WhatsApp, but this proved ineffective due to incomplete responses. Therefore, the research team conducted data collection in person using printed questionnaires, administered during direct home visits to families with children aged 6–24 months diagnosed with stunting. Trained healthcare workers, coordinated by the lead researcher, ensured accuracy, completeness, and consistency of the data.

### 2.4. Data Analysis

Data analysis was conducted using Structural Equation Modeling (SEM). Because the primary objective of this study was to test a theoretical model, we applied a covariance-based SEM approach implemented through Partial Least Squares (PLS). SEM-PLS was chosen as it is suitable for exploratory models, robust in handling non-normal data distributions, and appropriate for the relatively modest sample size (n = 324). Before applying SEM-PLS, collinearity analysis was performed by calculating the Variance Inflation Factor (VIF) for each indicator, with all values below the threshold of 5, indicating no multicollinearity issues. Convergent validity was assessed using Average Variance Extracted (AVE ≥ 0.50), while discriminant validity was examined through the Fornell–Larcker criterion and the Heterotrait–Monotrait ratio (HTMT < 0.85). Reliability was evaluated using composite reliability (CR > 0.70) and Cronbach’s alpha (>0.60). These steps ensured that the model met the requirements for hypothesis testing. All analyses were conducted using SmartPLS version 4.

## 3. Results

### 3.1. Demography Characteristics

Phase 1 of the study was conducted from 1 January 2025 to 1 May 2025. Questionnaires were distributed to respondents via WhatsApp using Google Forms, as well as through direct distribution, targeting a total population of 324 families with children experiencing stunting who met the inclusion criteria.

Based on the analysis presented in Table 1, among the 324 respondents, the majority of fathers were self-employed (204; 63%), while most mothers were unemployed (242; 74.7%). Regarding educational attainment, most fathers had completed senior high school (138; 42.6%), and the majority of mothers had also completed senior high school (162; 50.0%). In terms of birth order, most stunted children were second-born (130; 40.1%). Additionally, the majority of stunted children were male (179; 55.2%), and the highest proportion of stunted children were aged 17–24 months (155; 47.8%).

### 3.2. Description of Questionnaire Results

The study results were categorized into several factors: Demographic Factors (X1), measured through two indicators: Age (X1.1) and Environment (X1.2); Educational Factors (X2), measured by education level (X2.1) and knowledge (X2.2); Technological Factors (X3), including ownership of technology (X3.1) and utilization of technology (X3.2); Economic Factors (X4), assessed based on occupation (X4.1) and income (X4.2); Cultural Values (X5), measured through Family-Centered Care (X5.1), participation in posyandu (X5.2), and immunization practices (X5.3); Family Empowerment (X6); and Quality of Complementary Feeding (Y1).

A detailed description of these factors (X1–Y1) is presented in Supplementary Materials S3.

Based on the analysis, among 324 respondents, most mothers were aged 31–40 years (151; 46.6%) and lived in environments considered low-risk for stunting (298; 92%). The majority had completed senior high school (162; 50%) and demonstrated moderate knowledge (269; 83%). Almost all respondents owned technology (321; 99.1%), and most reported moderate use of technology (252; 77.8%). Most mothers were unemployed (242; 74.7%), and the majority of families had income categorized as low (238; 73.5%).

Regarding cultural values, most respondents reported inadequate Family-Centered Care (167; 51.5%), while the majority attended posyandu regularly (276; 85.2%) and had completed child immunizations (295; 91.0%). For family empowerment indicators, most respondents demonstrated good acceptance of healthcare workers (311; 96%), good acceptance of healthcare services (306; 94.4%), and good ability to identify and express problems (213; 65.7%). Furthermore, most respondents showed good ability in performing health care (189; 58.3%), moderate ability to utilize health facilities (204; 63.0%), moderate ability to implement preventive actions (228; 67.3%), and moderate ability to carry out health promotion activities (168; 51.9%).

Regarding complementary feeding practices, most respondents adhered to the recommended timing for introducing (311; 96.0%) and met the appropriate feeding frequency (196; 60.5%). However, dietary diversity was only partially achieved among respondents (158; 48.7%), and more than half (172; 53.1%) were classified in the high category for adequacy of complementary feeding practices.

### 3.3. Evaluation of the Outer Model

**Figure 1:**
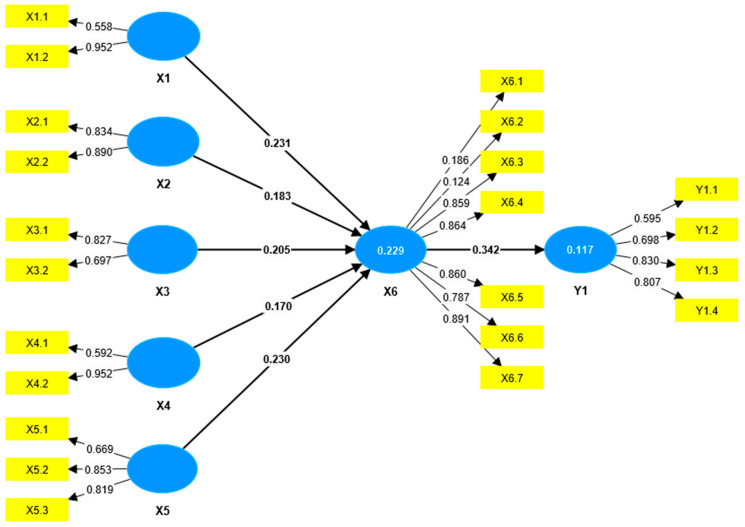
Outer Model Constructs.

The evaluation of the measurement model aims to assess the validity and reliability of each construct, which consists of Construct Validity Evaluation and Construct Reliability Evaluation (Figure 1).

Construct validity was assessed by calculating convergent validity. Convergent validity was determined through the loading factor and Average Variance Extracted (AVE) values. An instrument is considered to meet convergent validity if the loading factor is greater than 0.5 and the AVE exceeds 0.5. The analysis showed that two indicators had loading factor values below 0.5, namely X6.1 (Acceptance of Health Workers) and X6.2 (Acceptance of Health Services). Therefore, based on convergent validity, these indicators were deemed invalid in measuring their respective constructs and were removed (Table S8, Supplementary Materials S4).

After the removal of these indicators, all remaining indicators achieved loading factors and AVE values greater than 0.5, indicating that all indicators met the criteria for convergent validity (Table S9, Supplementary Materials S4).

In addition, discriminant validity was assessed using the Heterotrait–Monotrait ratio (HTMT). The HTMT values were found to be below 0.85, confirming that discriminant validity was satisfied. This indicates that each indicator accurately measured its corresponding latent variable (Table S10, Supplementary Materials S4).

Construct reliability was evaluated using Cronbach’s alpha and composite reliability. According to the criteria, a construct is considered reliable if the composite reliability exceeds 0.7 and Cronbach’s alpha exceeds 0.6. All variables demonstrated Cronbach’s alpha values above 0.6 and composite reliability values above 0.7, confirming that all constructs were reliable (Table S11, Supplementary Materials S4).

### 3.4. Evaluation of the Inner Model

The evaluation of the structural model, or inner model, involves assessing the goodness of fit, which includes the coefficient of determination (R^2^), predictive relevance, and hypothesis testing. The coefficient of determination (R^2^) is used to determine the extent to which endogenous variables can explain the variance of exogenous variables, or, in other words, to assess the contribution of exogenous variables to endogenous variables.

The R^2^ value for the Family Empowerment variable was 0.223 (22.3%). This indicates that the variability of Family Empowerment can be explained by Demographic Factors, Educational Factors, Technological Factors, Economic Factors, and Cultural Values by 22.3%. In other words, these five factors contribute 22.3% to Family Empowerment, while the remaining 77.7% is influenced by other variables not included in this study.

The R^2^ value for the Quality of Complementary Feeding variable was 0.115 (11.5%), suggesting that Family Empowerment explains 11.5% of the variability in quality. The remaining 88.5% is attributed to other variables outside the scope of this research.

The effect size (f^2^) measures the relative impact of an exogenous variable on an endogenous variable. According to Cohen (cited in Hair et al., 2022 [22]), effect size thresholds are 0.02 (small), 0.15 (medium), and 0.35 (large), with values below 0.02 indicating no measurable effect. The analysis showed that the largest effect size was from Family Empowerment (X6) on Quality (Y1), with an f^2^ value of 0.131, and categorized as small to medium. All other independent variables had effect sizes on Family Empowerment within the small-to-medium range (Table S12, Supplementary Materials S4).

**Figure 2:**
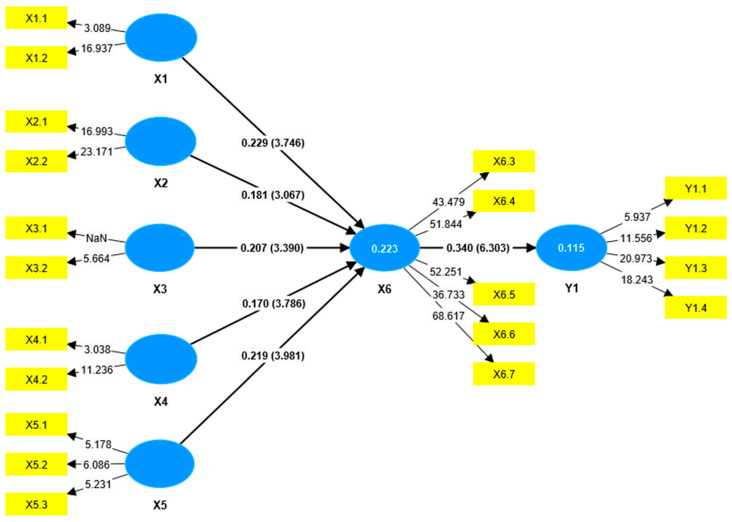
Inner Model Construct.

Hypothesis testing was conducted to examine the significance of the influence of exogenous variables on endogenous variables. The criteria state that if the T-statistic ≥ T-table (1.96) or *p*-value < α (0.05), then the effect is considered significant. The results of significance testing and the model are presented in the following figure (Figure 2) and tables.

The hypothesis testing results (Table 2) indicate that all exogenous variables—Demographic Factors (X1), Educational Factors (X2), Technological Factors (X3), Economic Factors (X4), and Cultural Values (X5)—had a significant positive effect on Family Empowerment (X6), with T-statistics greater than 1.96 and *p*-values less than 0.05. Among these, Demographic Factors (β = 0.229, *p* < 0.001) and Cultural Values (β = 0.219, *p* < 0.001) demonstrated the strongest influence, followed by Technological Factors (β = 0.207, *p* = 0.001), Educational Factors (β = 0.181, *p* = 0.002), and Economic Factors (β = 0.170, *p* < 0.001). Additionally, Family Empowerment (X6) significantly influenced the Quality of Complementary Feeding () (Y1) (β = 0.340, T = 6.303, *p* < 0.001), indicating that higher levels of family empowerment are associated with better complementary feeding practices.

The indirect effect analysis (Table 3) showed that all indirect paths yielded T-statistics greater than 1.96 and *p*-values less than 0.05, indicating statistical significance. These findings suggest that Demographic Factors (X1), Educational Factors (X2), Technological Factors (X3), Economic Factors (X4), and Cultural Values (X5) significantly influence the Quality of Complementary Feeding (Y1) through Family Empowerment (X6). In other words, Family Empowerment (X6) serves as a significant mediator in the relationship between these factors and complementary feeding quality.

## 4. Discussion

The study conducted on 324 mother–child pairs aged 6–24 months across 11 community health centers (puskesmas) in Kediri Regency demonstrated that improving the quality of complementary feeding as a strategy to prevent stunting in children aged 6–24 months using a transcultural care approach is influenced by several factors. These include demographic factors (age and environment), educational factors (maternal education level and knowledge), technological factors (ownership and utilization of technology), economic factors (occupation and income level), cultural values (family-centered care, participation in posyandu, and immunization), and family empowerment factors, particularly at the collaboration stage. The transcultural care dimension significantly impacts the quality of complementary feeding practices.

*Puskesmas* (Community Health Center) are primary healthcare facilities in Indonesia that provide both public health and individual health services, with a strong emphasis on promotive (health promotion) and preventive (disease prevention) efforts within their designated catchment area. On the other hand, Posyandu is a community-based health service in Indonesia that plays a key role in stunting prevention and early detection of child growth through monthly weight monitoring of children under five [23]. Its activities include growth monitoring, nutrition counseling, basic healthcare, family planning, and immunization, with professional healthcare workers delivering medical services, while community health volunteers (kader) conduct routine tasks such as weighing and measuring children [24].

A related study involving 145 mother–child pairs aged 12–36 months in the working area of Janti Community Health Center found that feeding patterns among stunted toddlers within a transcultural nursing framework are influenced by economic factors, regulations and policies, cultural values and lifestyle, religiosity and philosophy, and social and family support, as well as technology. Among these, cultural beliefs and lifestyle were the most critical factors affecting feeding practices. Therefore, healthcare providers are encouraged to intensify preventive and persuasive measures through health counseling for mothers and families [25].

Another study conducted at Kaluku Bodoa Community Health Center, Makassar, involving 103 children aged 24–59 months and using a transcultural nursing approach, reported a significant association between stunting and technological factors, social and family support, cultural values and lifestyle, economic factors, and educational factors, with social and family support being the most influential factor [26]. Similarly, research conducted in the working area of Betungan Community Health Center, Bengkulu, revealed correlations between stunting and technological factors, family and social support, and cultural values and lifestyle, as well as economic factors, whereas no correlation was found between educational characteristics and stunting [27].

Parental roles play a crucial part in child growth and development, particularly regarding decisions on feeding practices, exclusive breastfeeding, meal diversity, and maintaining hygiene within the household. Effective parental involvement is expected to improve nutritional status in children as a preventive measure against stunting, thereby emphasizing the necessity of family empowerment [28].

**Figure 3:**
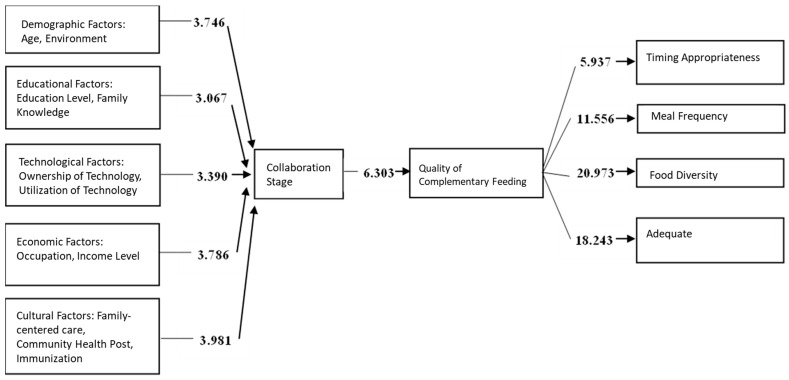
Research findings on the development of a family empowerment model based on transcultural care theory to improve the quality of complementary feeding as an effort to prevent stunting in children aged 6–24 months.

Based on the overall results of hypothesis testing, the significant pathways reflecting the proposed research model can be established (Figure 3).

The proposed model consists of demographic factors (age and environment), educational factors (education level and maternal knowledge), technological factors (ownership and utilization of technology), economic factors (occupation and income level), cultural values (shared family meals, posyandu participation, and immunization), and family empowerment (collaboration stage), all of which influence the quality of complementary feeding. The quality of complementary feeding should meet four key indicators: timely introduction of complementary foods, minimum meal frequency, minimum dietary diversity, and minimum acceptable diet. Meeting these indicators is expected to help prevent stunting in children aged 6–24 months.

Among the measured indicators, the highest contribution to demographic factors (X1) was from environment (loading factor = 0.958); for educational factors (X2), from maternal knowledge (loading factor = 0.888); for technological factors (X3), from technology ownership (loading factor = 0.783); for economic factors (X4), from income level (loading factor = 0.970); for cultural values (X5), from posyandu participation (loading factor = 0.829); and for quality (Y1), from dietary diversity (loading factor = 0.856).

This study shows that demographic, environmental, educational, technological, economic, and cultural factors shape family empowerment and complementary feeding quality in stunting prevention. Most mothers were aged 31–40 years, with stunted children often being first- or second-born, suggesting pregnancies at high-risk ages (>35 years). Interestingly, no mothers under 20 years were identified, despite the high rate of early marriage in Kediri [29], which may reflect that children from early marriages are being raised by other relatives. Environmental risks such as poor sanitation and limited clean water are widely recognized, yet their impact appeared less pronounced here, likely due to improved access supported by the *Sanitasi Total Berbasis Masyarakat (STBM)* program. Education and maternal knowledge also played an important role; most mothers had completed senior high school and showed adequate knowledge, differing from studies that found no significant association between education and stunting, which may be explained by contextual or methodological differences [30].

Technology ownership among mothers was high but utilization low, with only 6.2% responding to online questionnaires, reflecting barriers such as poor internet coverage and data costs. Nevertheless, technology remains a promising tool for nutrition education and early detection if access can be improved. Economic constraints were evident, with most families living below the minimum wage, restricting dietary diversity and healthcare access, and reinforcing poverty as a structural driver of stunting [31].

Cultural values, including paternal involvement in child-rearing, participation in *posyandu*, and completion of immunization schedules, play an important role in shaping the quality of complementary feeding and stunting prevention among children aged 6–24 months. In this study, the most prominent cultural factor was the limited implementation of family-centered care, particularly the practice of shared meals with fathers, while participation in *posyandu* and immunization coverage were generally high [32]. Paternal involvement—such as supporting healthy dietary practices, accompanying children to health facilities, and ensuring timely immunizations—can significantly strengthen efforts to meet children’s nutritional needs. Shared meals with fathers may enhance the effectiveness of nutritional care, increase children’s appetite, and contribute to stunting reduction. Importantly, father involvement in child feeding represents a simple, culturally adaptable practice that can be applied across families.

The predominance of fathers working in self-employment reflects household income that may be unstable or irregular, which can limit the family’s ability to consistently provide diverse and nutritious foods. At the same time, the high proportion of unemployed mothers, while reducing household income, may increase maternal availability for childcare and feeding, thereby influencing complementary feeding practices. These dynamics highlight how economic stability and caregiving roles directly shape family empowerment and the effectiveness of nutritional practices, aligning with the proposed model that emphasizes the interaction of economic and cultural factors in stunting prevention.

Importantly, most families demonstrated empowerment up to the collaboration stage, providing a strong basis for integrated interventions. Collaboration among health workers, volunteers, and families, supported by continuous training and recognition for *kader*, is essential to improve complementary feeding quality and reduce stunting.

Most existing models for stunting prevention in Indonesia and similar contexts have largely focused on biomedical interventions, socioeconomic support, or maternal education as the primary levers for improving child nutrition [11]. While these approaches are valuable, they often overlook the role of cultural values and family dynamics that strongly shape feeding practices. By integrating transcultural care theory into the family empowerment model, this study highlights that cultural practices—such as paternal involvement and shared meals—are not peripheral but central determinants of complementary feeding quality. This culturally sensitive perspective distinguishes our model from conventional frameworks, offering a more holistic approach that can be adapted to diverse communities where cultural norms significantly influence child nutrition.

A key strength of this study is the identification of cultural values, particularly the role of shared meals with fathers, as an important determinant of stunting prevention in children aged 6–24 months. This provides novel empirical evidence and offers policymakers a culturally sensitive approach that can be integrated into family-based nutrition programs. Additional strengths include the relatively large sample drawn from multiple community health centers, the use of transcultural care theory as a guiding framework, and the application of a validated questionnaire, which ensured a holistic and reliable assessment of demographic, educational, technological, economic, and cultural factors.

However, the study has several limitations. Its cross-sectional design precludes causal inference, and reliance on self-reported questionnaires may introduce reporting bias. Environmental conditions were assessed indirectly through self-reports rather than direct observation, which may not capture the full context. The sampling was conducted by a purposive sampling method that might affect the precision of our data. In addition, 95% confidence intervals for estimates were not calculated, which may restrict the precision of effect size interpretation. Future studies should report confidence intervals alongside beta coefficients and *p*-values to improve transparency. Finally, as the study was conducted in Kediri Regency, cultural practices may differ in other regions of Indonesia, limiting generalizability.

These findings have important implications for policy. In the Indonesian context, the integration of culturally grounded family empowerment strategies—such as promoting paternal involvement and shared meals—could be incorporated into the Ministry of Health’s national stunting prevention programs, alongside existing initiatives on nutrition education, sanitation, and community health volunteer (*kader*) engagement. At the policy level, this approach reinforces the need to design interventions that go beyond biomedical and socioeconomic determinants by explicitly addressing cultural values and family dynamics.

Beyond Indonesia, the proposed model provides a framework that can be adapted and tested in other regions or countries where cultural practices strongly influence child nutrition and caregiving. By contextualizing transcultural care principles to local traditions and family structures, the model can support global efforts to prevent stunting and promote child health through culturally sensitive and family-centered interventions.

## 5. Conclusions

This study demonstrates that a family empowerment model based on transcultural care theory can significantly improve the quality of complementary feeding and support stunting prevention in children aged 6–24 months. The model emphasizes the role of demographic, educational, technological, economic, and cultural factors, with particular importance placed on paternal involvement and culturally sensitive practices. Beyond Kediri Regency, this model can be adapted and tested in other regions or countries where cultural values strongly influence child nutrition, thereby broadening its applicability.

For policymakers and healthcare professionals, the findings highlight the need to complement existing health education and community-based programs with culturally grounded strategies that strengthen family involvement, including shared meals and equitable parenting roles. Future research should build on these findings by implementing and evaluating comprehensive, culturally grounded, and family-centered interventions in real-world community settings. Additionally, the next study should employ longitudinal or experimental designs to confirm causal relationships and assess the long-term effectiveness of this model in diverse community settings.

## Figures and Tables

**Table 1:** Demographic Characteristics of Respondents.

Indicator	Category	Percentage (n)
Father’s Occupation	Teacher	1.5% (5)
	Trader	16.4% (53)
	Employee	0.9% (3)
	Farmer	16.4% (53)
	Civil Servant	1.2% (4)
	Unemployed	0.6% (2)
	Entrepreneur	63.0% (204)
Mother’s Occupation	Teacher	2.5% (8)
	Trader	9.0% (29)
	Farmer	2.5% (8)
	Civil Servant	1.9% (6)
	Unemployed	74.7% (242)
	Entrepreneur	9.6% (31)
Father’s Education	Primary School	17.9% (58)
	Junior High	32.7% (106)
	Senior High	42.6% (138)
	Diploma	0.9% (3)
	Bachelor	5.6% (18)
	Master	0.3% (1)
Mother’s Education	Primary School	10.8% (35)
	Junior High	29.0% (94)
	Senior High	50.0% (162)
	Diploma	0.3% (1)
	Bachelor	9.9% (32)
Birth Order	1	39.2% (127)
	2	40.1% (130)
	3	18.5% (60)
	>3	2.2% (7)
Child’s Gender	Male	55.2% (179)
	Female	44.8% (145)
Child’s Age	6–12 months	25.0% (81)
	13–16 months	27.2% (88)
	17–24 months	47.8% (155)

**Table 2:** Results of Hypothesis Testing.

Effect	Coefficient	T Statistics (|O/STDEV|)	*p* Values
Demographic Factors (X1) → Family Empowerment (X6)	0.229	3.746	0.000
Educational Factors (X2) → Family Empowerment (X6)	0.181	3.067	0.002
Technological Factors (X3) → Family Empowerment (X6)	0.207	3.390	0.001
Economic Factors (X4) → Family Empowerment (X6)	0.170	3.786	0.000
Cultural Values (X5) → Family Empowerment (X6)	0.219	3.981	0.000
Family Empowerment (X6) → Complementary Feeding Quality (Y1)	0.340	6.303	0.000

**Table 3:** Indirect Effect Hypothesis Testing Results.

Indirect Effect	Coefficient	T Statistics (|O/STDEV|)	*p* Values
Demographic Factors (X1) → Family Empowerment (X6)	0.078	3.430	0.001
Educational Factors (X2) → Family Empowerment (X6)	0.061	2.826	0.005
Technological Factors (X3) → Family Empowerment (X6)	0.070	2.865	0.004
Economic Factors (X4) → Family Empowerment (X6)	0.058	3.266	0.001
Cultural Values (X5) → Family Empowerment (X6)	0.074	3.367	0.001

## Data Availability

All data are available within the manuscript and Supplementary Materials.

## Supplementary Materials

The following supporting information can be downloaded at: https://www.mdpi.com/article/10.3390/healthcare13172237/s1, Supplementary Materials S1. Sample Size Calculation; Supplementary Materials S2. Research Questionnaire; Supplementary Materials S3. Description of Research Results; Supplementary Materials S4. Outer Model Evaluation.
