# Supplementary material for: Determinants of Family Empowerment and Complementary Feeding Quality: Evidence from a Transcultural Care Framework
Source: Healthcare (Basel). 2025 Sep 8;13(17):2237. doi: 10.3390/healthcare13172237 (PMC12428396; doi:10.3390/healthcare13172237)
Supplement: Supplementary file 1 [file healthcare-13-02237-s001.zip › Supplementary Materials S1_English.pdf]

Sample Size Calculation

The required sample size was determined using the following formula:

$$\frac{N}{1 + N \cdot e^2}$$

Where:
n = Sample size
N = Population size
e = Margin of error

With a known population of 1,328 families, a 5% margin of error, and an alpha level of 0.05, the minimum required sample size was calculated as follows:

$$\frac{1328}{1 + (1328 \times 0.0025)}$$

n = 308 respondents
