# Supplementary material for: Determinants of Family Empowerment and Complementary Feeding Quality: Evidence from a Transcultural Care Framework
Source: Healthcare (Basel). 2025 Sep 8;13(17):2237. doi: 10.3390/healthcare13172237 (PMC12428396; doi:10.3390/healthcare13172237)
Supplement: Supplementary file 1 [file healthcare-13-02237-s001.zip › Supplementary Materials S2_English.pdf]

(TRANSLATED VERSION)

DEVELOPMENT OF FAMILY EMPOWERMENT MODEL BASED ON TRANSCULTURAL CARE THEORY TO IMPROVE THE QUALITY OF COMPLEMENTARY FEEDING (CF) IN THE PREVENTION OF STUNTING IN CHILDREN AGED 6–24 MONTHS IN KEDIRI REGENCY

QUESTIONNAIRE FILLING INSTRUCTIONS:

You are expected to fill out the questions below by placing a check mark (√) in the answer column that best reflects your current situation honestly.

1. All questions provided by the researcher should be answered.
2. If you have any questions that you do not understand, please ask the facilitator.

RESPONDENT DEMOGRAPHIC DATA

1) Code (Filled by researcher) :
2) Age : year
3) Father's occupation :
   - ☐ Enterpreneur ☐ Employee
   - ☐ Trader ☐ Civil Servant
   - ☐ Teacher ☐ Unemployed
4) Mother's occupation :
   - ☐ Enterpreneur ☐ Employee
   - ☐ Trader ☐ Civil Servant
   - ☐ Teacher ☐ Housewife
5) Father's education :
   - ☐ Primary ☐ Junior High
   - ☐ Senior High ☐ Diploma
   - ☐ Bachelor ☐ No School
6) Mother's education:
   - ☐ Primary ☐ Junior High
   - ☐ Senior High ☐ Diploma
   - ☐ Bachelor ☐ No School
7) Monthly family income:
   - ☐ < 500 thousand ☐ 3–4 million
   - ☐ 1–2 million ☐ > 4 juta million
   - ☐ 2–3 million
8) Does the family have any Health insurance (BPJS, KIS, others) ?
   - ☐ Yes
   - ☐ No

9) Does the family have a smartphone with internet access for stunting information?

- ☐ Yes
- ☐ No

10) Can the family access stunting information via smartphone?

- ☐ Yes
- ☐ No

11) Birth order of child with stunting:

- ☐ 1 ☐ 3
- ☐ 3 ☐ >3

12) Sex of child with stunting:

- ☐ Male ☐ Female

13) Age of child with stunting:

- ☐ 6-12 months ☐ 13-16 months
- ☐ 17-24 months

14) Can the family access clean water?

- ☐ Yes
- ☐ No

15) Does the family own toilet with enclosed space?

- ☐ Yes
- ☐ No

16) Does the family practice handwashing before feeding the child ?

- ☐ Yes
- ☐ No

17) How many times a day the child eats with the father?

- ☐ Never ☐ 2 times
- ☐ 1 time ☐ >3 times

18) How long does the child eat with the father? (Don't answer if the answer in point No.17 is "Never")

- ☐ 1-5 minutes ☐ 11-15 minutes
- ☐ 6-10 minutes ☐ more than 15 minutes

19) Is there any change in child's portion when eating with father? (Don't answer if the answer in point No.17 is "Never")

- ☐ Same ☐ ½ Portion
- ☐ ¼ Portion ☐ Always finished the whole portion

20) Does the child attend Posyandu for measuring weight and height ?

- ☐ Regularly ☐ Rarely
- ☐ Sometimes ☐ Never

21) Does the child received all the scheduled immunization according to age?

- ☐ Complete ☐ None
- ☐ Incomplete

Lampiran 4 KNOWLEDGE QUESTIONNAIRE

QUESTIONNAIRE FILLING INSTRUCTIONS:

You are expected to fill out the questions below by placing a check mark (√) in the answer column that best reflects your current situation honestly.

1. All questions provided by the researcher should be answered.
2. If you have any questions that you do not understand, please ask the facilitator.

Please place a check mark (√) in the available column and choose according to your actual condition.

Strongly Agree: SA

Agree: A

Neutral: N

Disagree: D

Strongly Disagree: SD

No	Statement	SA	A	N	D	SD
1	Stunting is a condition where a child suffers from long-term malnutrition, causing stunted growth.	5	4	3	2	1
2	Stunting in children can be prevented starting from proper pregnancy planning.	5	4	3	2	1
3	Stunting can be identified by measuring body weight alone.	5	4	3	2	1
4	One impact of stunting is that children become more susceptible to illness.	5	4	3	2	1
5	One risk factor for stunting in children is improper complementary feeding.	5	4	3	2	1
6	Regular attendance at Posyandu is one way of early detection of stunting.	5	4	3	2	1
7	Immunization according to the child's age is one method of stunting prevention.	5	4	3	2	1
8	Providing diverse protein sources is not a way to prevent stunting.	5	4	3	2	1
9	Complementary feeding can be given as early as 3 months of age.	5	4	3	2	1
10	Nutrition for children aged 0–6 months includes breast milk and bananas.	5	4	3	2	1
11	Breast milk can be given exclusively until 6 months and continued until 2 years.	5	4	3	2	1
12	Complementary feeding texture does not need to match the child's age.	5	4	3	2	1
13	Tofu and tempeh alone are sufficient protein sources for children.	5	4	3	2	1
14	Giving CF too early can cause intestinal blockage.	5	4	3	2	1
15	A sign that a child is not ready for texture progression is constipation.	5	4	3	2	1
16	An appropriate menu for a 7-month-old is soft rice + minced meat + vegetables + breast milk.	5	4	3	2	1

17	CF for 6–8 months: start with 2–3 spoons, gradually up to ½ bowl (150 ml).	5	4	3	2	1
18	CF for 6–8 months: mashed foods 3–4x + snack 1–2x + breast milk as often as possible.	5	4	3	2	1
19	Meal for 9–11 months: porridge + fish/chicken/egg/minced meat + fruit + breast milk.	5	4	3	2	1
20	Meal size for 9–11 months: start with ½ bowl, increase to ¾ bowl (350 ml).	5	4	3	2	1

Scoring:

Good: 76–100%

Fair: 56–75%

Poor: <55%

(ORIGINAL VERSION)

PENGEMBANGAN MODEL PEMBERDAYAAN KELUARGA BERBASIS TEORI *TRANSCULTURAL CARE* TERHADAP PENINGKATAN KUALITAS PEMBERIAN MAKANAN PENDAMPING ASI (MP-ASI) DALAM UPAYA PENCEGAHAN *STUNTING* PADA ANAK USIA 6-24 BULAN DI KABUPATEN KEDIRI

PETUNJUK PENGISIAN KUESIONER:

3. Bapak/Ibu diharapkan mengisi pertanyaan di bawah ini dengan cara memberikan tanda ***checklist*** **(√)** pada kolom jawaban yang dianggap sesuai dengan keadaan bapak ibu saat ini dengan jujur
4. Semua pertanyaan yang telah disediakan oleh peneliti diharapkan dijawab semuanya oleh bapak/ibu
5. Bila Bapak/Ibu memiliki pertanyaan yang tidak dimengerti silahkan bertanya pada fasilitator.

DATA DEMOGRAFI RESPONDEN

23) Kode (Diisi oleh peneliti) :

24) Usia : tahun

25) Pekerjaan ayah saat ini :

☐ Wiraswasta ☐ Pegawai
☐ Pedangang ☐ PNS
☐ Guru Tidak☐ekerja

26) Pekerjaan ibu saat ini :

☐ Wiraswasta ☐ Pegawai
☐ Pedangang ☐ PNS
☐ Guru Ibu R☐nah Tangga

27) Pendidikan terakhir ayah :

☐ SD ☐ SMP
☐ SMA ☐ Diploma
☐ Sarjana Tidak☐ekolah

28) Pendidikan terakhir ibu :

☐ SD ☐ SMP
☐ SMA ☐ Diploma
☐ Sarjana Tidak☐ekolah

29) Jumlah pendapatan keluarga setiap bulan:

☐ < 500 ribu ☐ 3–4 juta
☐ 1–2 juta ☐ > 4 juta
☐ 2–3 Juta

30) Apakah keluarga memiliki asuransi kesehatan seperti BPJS, KIS, atau asuransi kesehatan yang lain?

☐ Memiliki
☐ Tidak Memiliki

31) Apakah keluarga memiliki handphone yang dapat digunakan untuk mengakses internet dalam mencari informasi kesehatan mengenai *stunting*?

☐ Memiliki
☐ Tidak Memiliki

32) Apakah keluarga bisa mengakses internet dalam mencari informasi kesehatan mengenai *stunting*?

☐ Bisa
☐ Tidak Bisa

33) Anak keberapa yang mengalami stunting?

☐ 1 ☐ 3
☐ 3 ☐ >3

34) Apa jenis kelamin anak yang mengalami stunting ?

☐ Laki-Laki ☐ Perempuan

35) Berapa usia anak yang mengalami stunting?

☐ 6-12 bulan ☐ 13-16 bulan
☐ 17-24 bulan

36) Apakah dirumah bapak/ibu memiliki sumber air bersih?

☐ Ya
☐ Tidak

37) Apakah dirumah bapak/ibu memiliki tempat untuk BAK/BAB di ruang tertutup?

☐ Ya
☐ Tidak

38) Apakah bapak/ibu selalu menerapkan cuci tangan sebelum memberikan makanan kepada anak?

☐ Ya
☐ Tidak

39) Berapa kali anak makan dengan ayah dalam waktu sehari?

☐ Tidak pernah ☐ 2 kali
☐ 1 kali ☐ lebih dari 3 kali

40) Berapa lama durasi anak makan bersama dengan ayah dalam waktu sehari? (Tidak perlu dijawab jika soal no 17 tidak pernah)

☐ 1-5 Menit ☐ 11-15 Menit
☐ 6-10 Menit ☐ lebih dari 15 menit

41) Apakah ada perubahan porsi makan anak ketika makan bersama dengan ayah? (Tidak perlu dijawab jika soal no 17 tidak pernah)

☐ Sama saja ☐ Habis ½ Porsi
☐ Habis ¼ Porsi ☐ Makanan anak selalu habis

42) Apakah anak Bapak/Ibu rutin mengikuti kegiatan posyandu desa seperti timbang berat badan dan tinggi badan?

☐ Rutin ☐ Jarang
☐ Kadang-Kadang ☐ Tidak Pernah

43) Apakah anak bapak/ibu mendapatkan imunisasi sesuai dengan usianya saat ini?

☐ Imunisasi Lengkap ☐ Tidak Mengikuti Imunisasi
☐ Imunisasi Tidak Lengkap

Lampiran 4 Kuesioner Pengetahuan

PETUNJUK PENGISIAN KUESIONER:

1. Bapak/Ibu diharapkan mengisi pertanyaan di bawah ini dengan cara memberikan tanda ***checklist*** **(√)** pada kolom jawaban yang dianggap sesuai dengan keadaan bapak ibu saat ini dengan jujur
2. Semua pertanyaan yang telah disediakan oleh peneliti diharapkan dijawab semuanya oleh bapak/ibu
3. Bila Bapak/Ibu memiliki pertanyaan yang tidak dimengerti silahkan bertanya pada fasilitator.

Berilah tanda centang (√) pada kolom yang tersedia dan pilihlah sesuai keadaan yang sebenar-benarnya.

Sangat Setuju : SS
Setuju : S
Ragu-Ragu : R
Tidak Setuju : TS
Sangat Tidak Setuju : STS

**No**	**Pernyataan**	**SS**	**S**	**R**	**TS**	**STS**
1.	*Stunting* adalah kondisi dimana anak kekurangan gizi dalam jangka waktu lama sehingga anak menjadi kerdil	5	4	3	2	1
2.	*Stunting* pada anak dapat dicegah mulai dari perencanaan kehamilan yang matang	5	4	3	2	1
3.	*Stunting* dapat dikenali dengan cara mengukur berat badan badan saja	1	2	3	4	5
4.	Salah satu dampak yang ditimbulkan dari *stunting* adalah anak menjadi gampang sakit	5	4	3	2	1
5.	Salah satu faktor risiko penyebab *stunting* pada anak adalah pemberian makanan pendamping ASI yang salah	5	4	3	2	1
6.	Rutin mengikuti posyandu di desa merupakan salah satu cara deteksi dini *stunting* pada anak	5	4	3	2	1
7.	Imunisasi sesuai dengan usia anak merupakan salah satu pencegahan *stunting*	5	4	3	2	1
8.	Pemberian makanan beragam protein pada anak bukan merupakan salah satu cara pencegahan stunting pada anak	1	2	3	4	5
9.	Makanan pendamping ASI dapat diberikan sedini mungkin kepada anak ketika sudah berusia 3 bulan	1	2	3	4	5
10.	Jenis nutrisi yang dapat diberikan kepada anak usia 0-6 bulan adalah ASI dan pisang	1	2	3	4	5
11.	ASI (Air Susu Ibu) dapat diberikan kepada anak sampai usia 6 bulan kemudian dapat dilanjutkan hingga anak berusia 2 tahun	5	4	3	2	1
12.	Salah satu syarat utama pemberian Makanan Pendamping ASI pada anak adalah tekstur MP-ASI tidak harus sesuai dengan usia anak	1	2	3	4	5

13.	Tahu dan tempe saja sudah cukup memenuhi protein pada anak	1	2	3	4	5
14.	Risiko yang ditimbulkan ketika memberikan MP-ASI belum pada waktunya pada anak adalah terjadinya penyumbatan saluran cerna pada anak	5	4	3	2	1
15.	Cara mengetahui bahwa anak belum siap naik tekstur saat pemberian MP-ASI adalah anak menjadi susah buang air besar	5	4	3	2	1
16.	Contoh menu makanan yang tepat untuk anak usia 7 bulan adalah Nasi lembek + Daging cincang + Sayur + ASI	1	2	3	4	5
17.	Cara memberikan MP-ASI pertama kali untuk anak usia 6-8 bulan adalah dimulai dari 2-3 sendok penuh setiap makan kemudian ditingkatkan perlahan sampai setengah mangkuk berukuran 150 ml	1	2	3	4	5
18.	Cara pemberian MP-ASI untuk anak usia 6-8 bulan Makanan lumat 3-4 kali + snack (biskuit atau buah lumat) 1-2 kali + ASI sesering mungkin	5	4	3	2	1
19.	Komposisi makanan yang diberikan pada anak dengan usia 9-11 bulan adalah Nasi Tim atau Bubur Saring + Ikan/ayam/telur/daging cincang + Buah+ASI	5	4	3	2	1
20.	Ukuran pemberian MP-ASI pada anak usia 9-11 bulan adalah Mulai dari 1/2 mangkuk kemudian ditingkatkan perlahan sampai 3/4 mangkuk berukuran 350 ml	1	2	3	4	5

Skor Penilaian:

Kategori Baik dengan skor : 76-100%

Kategori Cukup dengan skor : 56-75%

Kategori Kurang dengan skor : <55%
