# Supplementary material for: Determinants of Family Empowerment and Complementary Feeding Quality: Evidence from a Transcultural Care Framework
Source: Healthcare (Basel). 2025 Sep 8;13(17):2237. doi: 10.3390/healthcare13172237 (PMC12428396; doi:10.3390/healthcare13172237)
Supplement: Supplementary file 1 [file healthcare-13-02237-s001.zip › Supplementary Materials S3_English.pdf]

Supplementary Material S3. Description of Research Results

Table S1. Description of Demographic Factors (X1)

Indicator	Category	Frequency (f)	Percentage (%)
X1.1 Mother's Age	21–30 years	138	42.6
	31–40 years	151	46.6
	41–50 years	35	10.8
X1.2 Environment	At risk of causing stunting	26	8.0
	Not at risk of causing stunting	298	92.0

Table S2. Description of Educational Factors (X2)

Indicator	Category	Frequency (f)	Percentage (%)
X2.1 Education Level	Elementary	35	10.8
	Junior High	94	29.0
	Senior High	162	50.0
	Bachelor	1	0.3
	Master	32	9.9
X2.2 Knowledge	Poor	18	5.6
	Adequate	269	83.0
	Good	37	11.4

Table S3. Description of Technological Factors (X3)

Indicator	Category	Frequency (f)	Percentage (%)
X3.1 Technology Ownership	Does not own	3	0.9
	Owns	321	99.1

X3.2 Technology Utilization	Poor	36	11.1
	Adequate	252	77.8
	Good	36	11.1

Table S4. Description of Economic Factors (X4)

Indicator	Category	Frequency (f)	Percentage (%)
X4.1 Occupation	Unemployed	242	74.7
	Teacher	8	2.5
	Trader/Farmer	37	11.4
	Self-employed	31	9.6
	Civil servant	6	1.9
X4.2 Income	Low	238	73.5
	Adequate	86	26.5

Table S5. Description of Cultural Values (X5)

Indicator	Category	Frequency (f)	Percentage (%)
X5.1 Family-centered care	Poor	167	51.5
	Adequate	114	35.2
	Good	43	13.3
X5.2 Posyandu	Rare	12	3.7
	Sometimes	36	11.1
	Regular	276	85.2
X5.3 Immunization	None	10	3.1
	Incomplete	19	5.9
	Complete	295	91.0

Table S6. Description of Family Empowerment (X6)

Indicator	Category	Frequency (f)	Percentage (%)
Receiving health workers	Poor	5	1.5
	Adequate	8	2.5
	Good	311	96.0
Receiving health services	Poor	4	1.2
	Adequate	14	4.3
	Good	306	94.4
Ability to identify and express problems	Poor	35	10.8
	Adequate	76	23.5
	Good	213	65.7
Practicing health care	Poor	25	7.7
	Adequate	110	34.0
	Good	189	58.3
Utilizing health facilities	Poor	36	11.1
	Adequate	204	63.0
	Good	84	25.9
Implementing preventive measures	Poor	37	11.4
	Adequate	218	67.3
	Good	69	21.3
Health promotion	Poor	31	9.6

activities			
	Adequate	168	51.9
	Good	125	38.6

Table S7. Description of Complementary Feeding (MP-ASI) Quality (Y1)

Indicator	Category	Frequency (f)	Percentage (%)
Y1.1 Timeliness of feeding	Not appropriate	13	4.0
	Appropriate	311	96.0
Y1.2 Feeding frequency	No	1	0.3
	Partial	127	39.2
	Yes	196	60.5
Y1.3 Food diversity	No	10	3.1
	Partial	158	48.7
	Yes	156	48.2
Y1.4 Adequacy of complementary feeding	Low	1	0.3
	Moderate	151	46.6
	High	172	53.1
