# Supplementary material for: Determinants of Family Empowerment and Complementary Feeding Quality: Evidence from a Transcultural Care Framework
Source: Healthcare (Basel). 2025 Sep 8;13(17):2237. doi: 10.3390/healthcare13172237 (PMC12428396; doi:10.3390/healthcare13172237)
Supplement: Supplementary file 1 [file healthcare-13-02237-s001.zip › Supplementary Materials S4_English.pdf]

Supplementary Material S4. Outer Model Evaluation

Table S8. Results of Convergent Validity Testing

Variable	Indicator	Factor Loading	AVE
X1 Demographic Factors	X1.1 Age	0.558	0.609
	X1.2 Environment	0.952	
X2 Educational Factors	X2.1 Education Level	0.834	0.744
	X2.2 Knowledge	0.890	
X3 Technological Factors	X3.1 Technology Ownership	0.827	0.585
	X3.2 Technology Utilization	0.697	
X4 Economic Factors	X4.1 Occupation	0.592	0.628
	X4.2 Income	0.952	
X5 Cultural Values	X5.1 Family-centered care	0.669	0.615
	X5.2 Posyandu	0.853	
	X5.3 Immunization	0.819	
X6 Family Empowerment	X6.1 Receiving health workers	0.186	0.527
	X6.2 Receiving health services	0.124	
	X6.3 Ability to identify and express problems	0.859	
	X6.4 Practicing health care	0.864	

	X6.5 Utilizing facilities	0.860	
	X6.6 Implementing preventive measures	0.787	
	X6.7 Promotive actions	0.891	
Y1 Complementary Feeding Quality	Y1.1 Timeliness of feeding	0.595	0.545
	Y1.2 Feeding frequency	0.698	
	Y1.3 Food diversity	0.830	
	Y1.4 Adequacy of complementary feeding	0.807	

Table S9. Results of Convergent Validity Testing After Reduction

Variable	Indicator	Factor Loading	AVE
X1 Demographic Factors	X1.1 Age	0.539	0.605
	X1.2 Environment	0.959	
X2 Educational Factors	X2.1 Education Level	0.835	0.744
	X2.2 Knowledge	0.890	
X3 Technological Factors	X3.1 Technology Ownership	0.821	0.585
	X3.2 Technology Utilization	0.704	
X4 Economic Factors	X4.1 Occupation	0.632	0.636
	X4.2 Income	0.935	
X5 Cultural Values	X5.1 Family-	0.674	0.615

	centered care		
	X5.2 Posyandu	0.849	
	X5.3 Immunization	0.818	
X6 Family Empowerment	X6.3 Ability to identify and express problems	0.854	0.734
	X6.4 Practicing health care	0.867	
	X6.5 Utilizing facilities	0.866	
	X6.6 Implementing preventive measures	0.797	
	X6.7 Promotive actions	0.897	
Y1 Complementary Feeding Quality	Y1.1 Timeliness of feeding	0.601	0.545
	Y1.2 Feeding frequency	0.694	
	Y1.3 Food diversity	0.830	
	Y1.4 Adequacy of complementary feeding	0.803	

Table S10. Results of Discriminant Validity Testing with HTMT

Indicator	X1	X2	X3	X4	X5	X6	Y1
X1	–						
X2	0.203	–					
X3	0.303	0.339	–				
X4	0.263	0.342	0.280	–			
X5	0.097	0.101	0.117	0.370	–		

X6	0.339	0.318	0.459	0.224	0.246	–	
Y1	0.305	0.107	0.319	0.197	0.398	0.415	–

Table S11. Results of Construct Reliability Testing

Variable	Cronbach's Alpha	Composite Reliability
Demographic Factors	0.435	0.740
Educational Factors	0.659	0.853
Technological Factors	0.295	0.737
Economic Factors	0.480	0.771
Cultural Values	0.680	0.826
Family Empowerment	0.909	0.932
Complementary Feeding Quality	0.713	0.825

Table S12. Effect Size (f^2) of Exogenous Variables on Endogenous Variables

Effect	f^2
Demographic Factors (X1) → Family Empowerment (X6)	0.066
Educational Factors (X2) → Family Empowerment (X6)	0.039
Technological Factors (X3) → Family Empowerment (X6)	0.053
Economic Factors (X4) → Family Empowerment (X6)	0.033
Cultural Values (X5) → Family Empowerment (X6)	0.059
Family Empowerment (X6) → Complementary Feeding Quality (Y1)	0.131
